# The relationship between social participation and cognitive function early after surgery of glioma patients

**DOI:** 10.1371/journal.pone.0319220

**Published:** 2025-02-25

**Authors:** Yinglian Xiao, Jing Tan, Guo Cheng, Yanhong Deng

**Affiliations:** Department of Neurosurgery, Chongqing University Cancer Hospital, Chongqing, China; Keio University School of Medicine Graduate School of Medicine: Keio Gijuku Daigaku Igakubu Daigakuin Igaku Kenkyuka, JAPAN

## Abstract

**Objective:**

Social Participation (SP) is known to benefit cognitive function. However, whether this positive relationship holds across the people with glioma has not been studied. The present study aimed to investigate the current status of SP and cognitive function of patients early after surgery with glioma, and therefore, explore the associations between cognitive function and SP.

**Methods:**

This study included 179 postoperative patients with gliomas within 6 months of surgery. Cognitive functioning was measured by the Montreal Cognitive Assessment (MoCA) including orientation, attention, learning and memory, executive functioning and verbal fluency. Social participation was obtained also by questionnaire survey with the Impact on Participation and Autonomy Questionnaire (IPA). The Pearson correlation analysis was used to explore the relation between cognitive functioning and social participation, and the efficacy of social participation in predicting cognitive functioning was evaluated using the receiver operating characteristic (ROC).

**Results:**

The prevalence of cognitive impairment in early postoperative glioma patients was 77.65%. The mean level of social participation was 37.96 ± 26.85 points, with poorer scores in the autonomy participation of family role and outdoor engagement dimensions. Patients’ cognitive functioning was positively correlated with social participation (*r* = −0.64, *P* < 0.001). And The areas under the ROC curve for social participation predicting cognitive function were 0.884.

**Conclusion:**

Early post-operative cognitive impairment was more common in glioma patients, which was positively correlated with social participation. Social rehabilitation programs for glioma patients should be actively constructed to promote the social participation of patients early after surgery in order to protect their cognitive function.

## 1. Introduction

Glioma is a malignant intracranial neoplasm, accounting for 40–50% of all cranial brain tumors [1], and exhibits an annual incidence rate of 5–6 cases per 100,000 individuals globally [2]. Gliomas exhibit high incidence of disability. Evidence shows that about 60% of glioma patients still have cognitive dysfunction after surgery due to the effects of Surgical physiological stress, radiotherapy, or long-term nerve compression [3–5]. This can have a significant impact on their postoperative survival [6]. Along with the improvements in survival with better treatment strategies, there has been a focus on the preservation of cognitive abilities. Studies show that the six-month period following surgery, often referred to as the early recovery phase, is critical for neurological recovery in patients with brain tumors [7]. Therefore, it is important to further investigate the protection of cognitive function and the factors involved in glioma patients early after surgery.

Age, education, tumor location and volume, extent of edema, Karnofsky Performance Score (KPS), adjuvant therapies and higher grade have been linked to glioma–related cognitive impairment [8–10]. The majority of these factors, however, are likely to be immutable and fixed, making it unlikely that interventions can be implemented to improve patients’ cognitive function. Cognitive rehabilitation is considered a proven strategy for addressing cognitive deficits in stroke and traumatic brain injury populations [11,12]. Physicians believe that social engagement is an important factor influencing the success of cognitive rehabilitation interventions [13]. However, there is still a lack of evidence regarding key factors in the current practice of cognitive rehabilitation for glioma patients. Further research is still needed to confirm how social engagement plays a role in cognitive rehabilitation among patients with glioma.

Social participation is a series of activities in which individuals participate in social interactions to achieve their personal goals and values, including political, cultural, economic and public welfare activities. Research has found that different types of social engagement can slow down cognitive decline in the brain of normal population [14]. The effects of brain tumors and treatments (e.g., weakness, paralysis, fatigue and epilepsy) may impair an individual’s ability to maintain relationships, fulfil social roles and participate in valued social groups [15]. Although it is well known that patients with brain tumors experience high levels of dysfunction(including cognitive dysfunction) and social decline [16,17], the relationship between social engagement and cognitive recovery in postoperative gliomas remains unclear. Therefore, our study was designed to investigate the correlation between cognitive functioning and the social engagement in glioma patients in the early postoperative period, and to explore the extent to which levels of social engagement can serve as predictors of cognitive functioning.

## 2. Materials and methods

### 2.1. Study design

We conducted a cross-sectional investigation of cognitive function and social participation in patients with glioma who were admitted to a tertiary specialized hospital in Chongqing, China from August 1, 2022 to November 31, 2023.

### 2.2. Sampling and inclusion criteria

The sample size was calculated by PASS15 software (NCSS, Kaysville, Utah, USA), using the following formula: *n* = Z^2^ × *p* × (1 − *p*)/*d*^2^. *n* is the sample size, Z = 1.96, *p* is the prevalence of glioma, and according to the research before, *p* = 6.4/100,000 [18], *d* = 0.02 was the acceptable margin of error, taken as 0.25 times of the standard deviation of cognition level with glioma patients [19]. The final calculated sample size is 91, and given that 20% of the questionnaires were invalid, at least 114 patients were needed in this study. The total sample size for our final survey was 179.

Patients that met the inclusion criteria were included in our study: ①patients with pathologically diagnosed primary glioma. ② age ≥ 18 years. ③ patients who were conscious and answerable with the ability to read and understand. ④ patients with no history of medical conditions that would cause cognitive dysfunction (Alzheimer’s disease, dementia, Parkinson’s disease, etc.). ⑤ ≤ 6 months after surgery (The time period was specified based on prior literature [7] and clinical experience). ⑥ Patients and their families agreed to participate in the study and signed informed consent.

Exclusion criteria were as below: ① patients with a history of drug addiction, long-term alcoholism, history of schizophrenia, severe anxiety, depression and other psychiatric disorders and family history. ②those with other malignant tumors.

### 2.3. Ethical considerations

This study was performed in line with the principles of the Declaration of Helsinki. Approval was granted by the Hospital Ethics Committee (CZLS2023146-A) and filed with the China Medical Research Registration and Filing Information System (MR-50-23-023000). All respondents signed a written informed consent form.

### 2.4. Measures

#### 2.4.1. General information.

A general information questionnaire was designed by the researcher through literature review. Which included age, education level, place of residence, marital status, the main payment method for medical expenses, the grade and location of the tumor, and whether or not they received radiotherapy, etc.

#### 2.4.2. Cognitive functions.

Patients’ Cognitive functioning was measured by the Montreal Cognitive Assessment (MoCA). MoCA is a widely used and rapid screening instrument for the evaluation of mild cognitive impairment. Most of the articles showed MoCA to be superior to MMSE in discriminating between individuals with mild cognitive impairment and no cognitive impairment [20]. It assesses different cognitive domains include visuospatial and executive skills, attention and concentration, memory, language, conceptual thinking [21]. The total MoCA score is in a range of 0 to 30 points with higher scores denoting better cognitive functioning. And a score of 26 or above is considered normal.

#### 2.4.3. Social participation.

The Impact on Participation and Autonomy Questionnaire (IPA) developed by Dutch researchers Cardol et al [22], was used to measure patients’ perceived participation and autonomy. The Chinese version [23] contains 4 dimensions, including indoor autonomy, family role autonomy, outdoor autonomy, and social life and social relationships, with a total of 25 items. A 5-point Likert scale was adopted, with each item scoring 0–4 points from “fully compliant” to “not at all compliant”, and the total score ranging from 0–100 points, with higher scores indicating poorer levels of social participation. The Cronbach’s α for this scale was 0.976 [24].

### 2.5. Data collection

Disease-related information was completed by reviewing medical records, and the rest of the information was filled out in a one-on-one question-and-answer session with the researcher and patient. Each researcher was trained to administer the questionnaires before investigation. The purpose and importance of this study were explained to patients, and their informed consent was obtained. To ensure credibility, the participants were provided with a quiet private room to fulfil the questionnaires. According to the questions narrated by the researcher, the patients were asked to give verbal answers, written answers, or motorized feedback, and the whole survey took 30–45 min. The data were double-checked for accuracy and then entered into EpiData 3.1.

### 2.6. Statistical analysis

SPSS 25.0 software was used for data analysis. Independent-sample t-tests and one-way analysis of variance (ANOVA) were used to conduct univariate analyses for demographic and clinical characteristics. The correlation between cognitive function and social participation scores was analyzed. Pearson correlation analysis was used if the data followed a normal distribution, and Spearman correlation analysis was used if the data did not follow a normal distribution. The predictive efficacy of social participation on cognitive function was evaluated by the Receiver Operating Characteristic (ROC), and the multiple linear regression analysis was used to identify respectively the effect of the various variables on cognitive function. A significance level of *P* <  0.05 was deemed indicative of statistical significance.

## 3. Results

### 3.1. Cognitive function in patients with different characteristics

Of the 183 patients initially recruited, 4 declined to participate because of an inability to complete the questionnaire as a consequence of poor health. Therefore, a total of 179 patients (M/F, 99/80) completed the questionnaires. The average age of the participants was (46.46 ± 13.78) years, ranged from 18 ~  73 years. About half of the patients (53.1%) were frontal lobe occupied, and 65.9% of them were WHO tumor classifications of grades IV. In addition, 29.1% of the participants had epilepsy and 24.0% of them had cerebral edema occurring in the postoperative period, and a total of 64.2% of the participants in this study having undergone adjuvant treatments such as radiotherapy, chemotherapy, simultaneous radiotherapy, chemotherapy, or targeted therapies after surgery. Complete demographic and clinical data are shown in Table 1.

**Table 1:** Cognitive function in patients with different characteristics (*n* =  179).

Variables	*n*(%)	MOCA(M ± SD)	*r/F/t*	*P*
**Age, M ± SD, y**	46.46 ± 13.78	18.77 ± 8.25	−0.434	<0.001
**Gender**				
Male	99 (55.3)	18.09 ± 9.04	−1.260	0.209
Female	80 (44.7)	19.61 ± 7.11		
**Marital Status**				
Single	18 (10.1)	24.83 ± 4.38	7.606	0.001
Married	142 (79.3)	18.53 ± 7.99		
Divorced/Widowed	19 (10.6)	14.84 ± 5.07		
**Education**				
Illiteracy	23 (12.8)	15.74 ± 6.88	7.246	<0.001
Primary school	33 (18.4)	14.18 ± 8.37		
Secondary school	57 (31.8)	21.11 ± 6.40		
High school	32 (17.9)	22.63 ± 7.50		
Junior college or higher	34 (19.0)	17.74 ± 9.63		
**Residence**				
City and towns	55 (30.7)	16.84 ± 9.63	−1.913	0.059
Rural area	124 (69.3)	19.63 ± 7.44		
**Self-care ability**				
Fully self-care	54 (30.2)	23.72 ± 4.95	39.237	<0.001
Partially self-care	96 (53.6)	18.74 ± 7.35		
Depend entirely on others	29 (16.2)	9.66 ± 3.33		
**Epilepsy**				
Yes	52(29.1)	18.00 ± 7.49	0.672	0.425
No	127(70.9)	19.09 ± 8.55		
**Cerebral edema**				
Yes	43(24.0)	11.67 ± 8.29	−6.699	<0.001
No	136(76.0)	21.01 ± 6.87		
**Cancer stage**				
Stage Ⅱ	24 (13.4)	22.67 ± 7.06	3.367	0.037
Stage Ⅲ	37 (20.7)	18.89 ± 8.16		
Stage Ⅳ	118 (65.9)	17.94 ± 8.33		
**Cancer site**				
Frontal lobe	95 (53.1)	20.29 ± 7.52	6.952	<0.001
Temporal lobe	33 (18.4)	12.30 ± 4.30		
Parietal lobe	15 (8.4)	17.73 ± 4.37		
Occipital lobe	12 (6.7)	21.25 ± 3.98		
Cerebellum	11 (6.1)	17.82 ± 7.72		
Others	13 (7.3)	23.77 ± 5.83		
**Tumor lateralization**				
Left	72 (40.2)	16.11 ± 9.69	6.720	0.002
Right	81 (45.3)	20.70 ± 6.23		
Bilateral	26 (14.5)	20.12 ± 7.69		
**KPS score(mean±SD)**	81.96 ± 20.09	18.77 ± 8.25	0.601	<0.001
**Discomfort symptoms** ^a^				
No	55(30.7)	23.76 ± 4.71	34.585	<0.001
Yes	124(69.3)	16.56 ± 3.52		
**Adjuvant therapy**				
No	64(35.8)	17.27 ± 5.20	4.591	0.002
Radiotherapy	5(2.7)	23.00 ± 8.86		
Chemotherapy	12(6.7)	24.00 ± 4.00		
CCRT	85(47.5)	19.86 ± 7.76		
Others	13(7.3)	12.62 ± 7.28		

Abbreviation: KPS, Karnofsky Performance Status. CCRT, concurrent chemoradiotherapy.

^a^vomiting, fatigue, dizziness, headache, visual impairment, muscular weakness of the limbs.

The results of the univariate analysis showed that age, marital Status, education level, self-care ability, KPS score, cancer stage, cancer site, tumor lateralization, adjuvant therapy, cerebral edema and discomfort symptoms had statistically significant associations with cognitive function in glioma patients (*P* < 0.05), indicating that they were potential factors influencing cognitive function. Complete univariate analysis data are shown in Table 1.

### 3.2. Cognitive function

The total cognitive function score (MOCA) of glioma patients early after surgery ranged from 0 to 30, with an average score of 18.77 ± 8.25 points. 139 (77.65%) participants were classified as cognitive impairment (MOCA scores < 26 points). The MoCA total score and scores for each dimension are shown in Table 2.

**Table 2:** Cognitive function in glioma patients early after surgery (*n* =  179).

Variables	Total score (M ± SD)	<26 points(*n,%*)
**MoCA**	18.77 ± 8.25	139,77.65
Visuospatial and executive functioning	2.29 ± 0.82	
Naming	2.33 ± 0.84	
Attention	4.71 ± 1.85	
Language	1.72 ± 0.60	
Abstraction	1.23 ± 0.54	
Memory	1.76 ± 0.61	
Orientation	4.73 ± 1.89	

### 3.3. Social participation

The average scores of participants’ IPA and the dimensions, include indoor participation, family role participation, outdoor participation, social life and social relations were (37.96 ± 26.85), (8.01 ± 3.11), (14.15 ± 6.99), (8.59 ± 3.98), (7.21 ± 3.92), respectively, with higher scores on family role and outdoor autonomous participation, which is detailed shown in Table 3.

**Table 3:** Social participation in glioma patients early after surgery (*n* =  179).

Variables	Item	Score range	Total score (M ± SD)	Score Per Item (M ± SD)
**IPA**	25	0–98	37.96 ± 26.85	
Indoor participation	7	0–28	8.01 ± 3.11	1.14 ± 0.37
Family role participation	7	0–28	14.15 ± 6.99	2.02 ± 0.43
Outdoor participation	5	0–28	8.59 ± 3.98	1.72 ± 0.38
Social life and relations	6	0–20	7.21 ± 3.92	1.20 ± 0.11

### 3.4. Correlation between social participation and cognitive function

**Fig 1:**
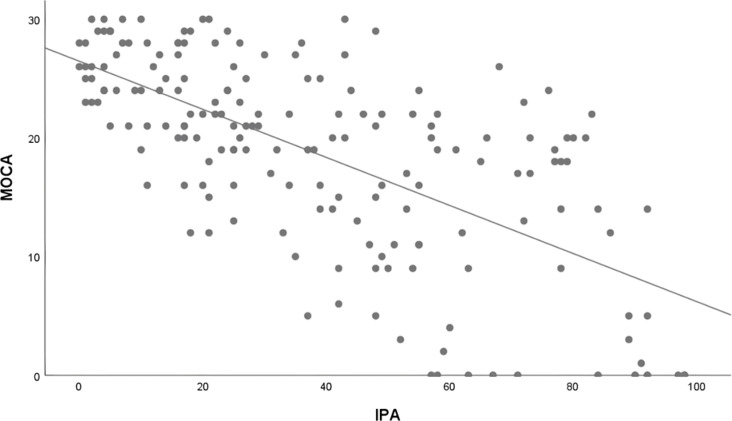
The scatter plot of IPA and MOCA scores.

The social participation and cognitive function scores followed a normal distribution, and partial correlation was used to analyze the correlation between them, with age, cancer stage and adjuvant therapy as covariates. The results showed that the scores of social participation was negatively correlated with the scores of cognitive function (*r* =  −0.64, *P* < 0.001), as shown in Table 4. The better social participation is (the scale is reverse scored), the better the cognitive function. The scatter plot of IPA and MOCA scores is shown in Fig 1.

**Table 4:** The relationship between social participation and cognitive function in glioma patients early after surgery (*n* = 179).

Variables	IPA	Indoor autonomy participation	Family role autonomy participation	Outdoor autonomy participation	Social life and relations
**MoCA**	−0.64***	−0.57***	−0.64***	−0.62***	−0.47***
Visuospatial and executive functioning	−0.56***	−0.50***	−0.58***	−0.56***	−0.36***
Naming	−0.51***	−0.49***	−0.48***	−0.50***	−0.35***
Attention	−0.45***	−0.39***	−0.44***	−0.44***	−0.37***
Language	−0.44***	−0.39***	−0.47***	−0.43***	−0.30***
Abstraction	−0.44***	−0.35***	−0.47***	−0.43***	−0.37***
Memory	−0.46***	−0.42***	−0.47***	−0.44***	−0.33***
Orientation	−0.55***	−0.50***	−0.53***	−0.53***	−0.43***

Notes: The data in the table are partial correlation coefficients (*r*), with age, cancer stage and adjuvant therapy as covariates.

***: *p* < 0.001

**Fig 2:**
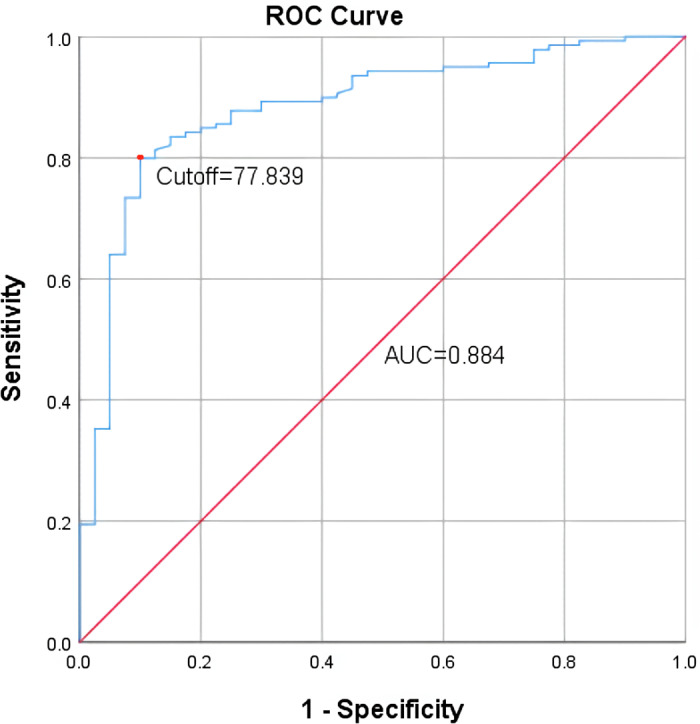
ROC curves for social participation in predicting cognitive function in glioma patients. Note. With age, cancer stage and adjuvant therapy as covariates.

The Receiver Operating Characteristic (ROC) curve was used to test the predictive power of social participation on cognitive function in glioma patients early after surgery, with age, cancer stage and adjuvant therapy as covariates. In our analysis, the ROC curve had an AUC of 0.884 [95% CI (0.825, 0.943), *P* < 0.001], indicating good discrimination ability. The optimal cut-off point was found at 77.839, with a corresponding sensitivity of 79.9% and specificity of 90%, and the Youden Index was 0.699. The ROC curve is shown in Fig 2.

### 3.5. Factors influencing cognitive function

The stepwise multiple linear regression (MLR) analysis indicated that the age, IPA, tumor lateralization, KPS, education level, self-care ability and cerebral edema significantly influenced cognitive function (*P* < 0.05). In this analysis, age, cancer stage, and adjuvant therapy were included as covariates using the “Enter” method, while the remaining variables were entered into the regression equation using the “Stepwise” method. The complete MLR analysis data are displayed in Table 5.

**Table 5:** Multiple linear regression analysis of cognitive function in glioma patients early after surgery (*n* =  179).

Variables	Unstandardized Coefficients		Standardized Coefficients	*t*	*P*
	*β*	Std. Error	*β*’		
**Constant**	8.811	5.510		1.599	0.112
**Age**	−0.151	0.031	−0.253	−4.842	<0.001
**Adjuvant therapy**	0.065	0.815	0.004	0.079	0.937
**Cancer stage**	0.954	0.559	0.083	1.706	0.090
**IPA**	−0.098	0.027	−0.319	−3.588	<0.001
**Tumor lateralization**	2.413	0.580	0.204	4.157	<0.001
**KPS**	0.275	0.021	0.670	12.844	0.010
**Education**	1.030	0.361	0.160	2.851	0.001
**Self-care ability**	−1.789	0.758	−0.145	−2.360	0.019
**Cerebral edema**	2.426	1.039	0.126	2.335	0.021

The predictive model of cognitive function constructed by multiple liner regression *F* =  38.925, *P* < 0.001, *R*^*2*^ = 0.675, Adjusted *R*^*2*^ =  0.657.

## 4. Discussion

Cognitive decline is a primary contributor to disability and reduced autonomy, significantly impacting quality of life and leading to significant burden to economic and social systems [6]. The prevalence of cognitive deficits among glioma patients ranges from 30% to 80%, and in this study was 77.65%, contingent upon the specific type of cognitive impairment, the assessment tool utilized, and the timing of the evaluation [25,26]. Furthermore, these deficits correlate with various factors, including age, tumor volume, tumor location, tumor grade, the Karnofsky Performance Status (KPS) score, radiotherapy, and additional variables [8]. Notably, cognitive impairment can also be observed in patients with low-grade gliomas [27].

Cognitive deficits observed in glioma patients during the early postoperative period predominantly manifested in delayed memory, visuospatial and executive functions, language, abstract thinking, attention, and computational skills. These impairments may be attributed to the fact that 71.5% of the patients in this study had lesions located in the frontal and temporal lobes. These brain regions are primarily responsible for organizing and executing learned behaviors, controlling verbal expressions, auditory perception, language reception, and visual memory, among other functions [28]. Meanwhile, 54.7% of the patients in this study exhibited lesions in the left cerebral hemisphere, while 86.6% were diagnosed with high-grade gliomas. Studies observed that individuals with gliomas situated in the left hemisphere demonstrated more pronounced cognitive dysfunction relative to those with gliomas in the right hemisphere [29]. Additionally, patients with high-grade gliomas experienced greater challenges in language acquisition, information processing speed, executive functioning, and speech compared to those with low-grade tumors [30].

Social neuroscience research suggests that engaging in valuable social activities can mitigate health threats for people with chronic conditions [15]. It has been found that patients with brain tumors frequently reduce their engagement in meaningful activities as the disease recurs or progresses [31]. Poudel’s study further reveals that survivors of central nervous system tumors experience a heightened sense of isolation relative to survivors of other cancers [32], and they also exhibit poorer peer relationships compared to the general population [33]. 73% of brain tumor patients experience significant psychosocial burden immediately following surgery [34]. Given that this burden may be attributed to both the diagnosis of the brain tumor and the stress related to surgical intervention, social support is likely to be especially crucial during the initial phase of treatment.

The early postoperative social participation score of glioma patients in this study was 37.96 ± 26.85. Notably, the lowest levels of participation were observed in autonomous family roles and outdoor activities. This finding may be attributed to the fact that 97.8% of the patients were residing with caregivers post-surgery, and only 30.2% of the patients achieved complete autonomy in their daily lives. Consequently, limitations in performing routine and/or strenuous household tasks were noted, indicating a necessity for assistance from caregivers, family members, and/or neighbors. CNS tumors can cause psychosocial problems following surgery. Indeed, due to surgical complications and symptoms associated with glioma, including seizures, fatigue, memory deficits, and communication difficulties, some patients may encounter deficits that lead to social or professional isolation [35,36]. Furthermore, the extent of social participation among patients early after sugery tends to diminish to varying degrees. This decline is attributable to the rigorous early postoperative regimen of radiotherapy, chemotherapy, and other therapeutic interventions, which restricts both the scope and duration of patients’ social interactions [8,37].

In conclusion, patients with glioma exhibit poor social quality early after sugery, which can be manifested in the form of limited socialization in the domains of physical activity, peer relationships, work and family life, which is not only interfered with by the patient’s symptom burden, but also in turn affects the patient’s symptom perception, and consequently, the quality of survival. These findings will inform the design of future interventions aimed at enhancing social engagement early after sugery for glioma patients. Specifically, we suggest the development of tailored interventions to support activities across varying levels of symptom burden. It is imperative to explore novel activities that can engage a greater number of postoperative glioma patients, thereby alleviating the constraints imposed by their condition. Additionally, diversifying the forms of social activities in constrained settings and improving the accessibility of technology-based interventions are crucial steps toward creating supportive social environments conducive to the well-being of glioma survivors.

Social determinants are receiving increasing attention as potential risk factors for cognitive decline [14,38]. Several studies have indicated that elevated levels of social engagement may prevent or decelerate cognitive decline in older populations [31,39,40]. However, there is a paucity of evidence concerning the relationship between social engagement and cognitive decline specifically attributable to disease processes in individuals diagnosed with glioma. Da Silva FC found that social participation especially exercise programs significantly improve cognitive function, processing speed, attention, and mental flexibility in patients with mild to moderate Parkinson’s disease [41]. The findings of the present study found that the degree of social participation among glioma patients can serve as a predictor of cognitive functioning status early after sugery. Specifically, a lower level of social participation during this period is associated with reduced cognitive functioning scores. The potential benefits of positive social relationships include increased mental stimulation, strategic thinking, neural growth, as well as better synaptic density [40]. Conversely, both objective and subjective social isolation may result in heightened stress reactivity, disrupt feedback inhibition within the adrenocortical axis, and subsequently cause neuronal damage in the prefrontal cortex and hippocampus, which are regions critical for learning and memory functions.

Changes in cognitive functioning, physical impairment, and emotional distress may lead to significant dependence on pre-existing social networks in glioma patients [42], most commonly on family support systems, affecting interpersonal relationships and reducing social participation other than family [15]. Therefore, it is recommended that healthcare professionals should focus on early assessment of the level of social participation in postoperative glioma patients, and targeted rehabilitation programs and necessary health guidance, including strengthening social participation and maintaining emotionally supportive relationships, may help to prevent cognitive decline and reduce the risk of improving patients’ quality of life.

## 5. Strengths and limitations

To the best of our knowledge, this study is the first to examine the relationship between social engagement and cognitive functioning in glioma patients early after surgery. Considering the substantial disease burden experienced by glioma patients, our findings hold significant public health implications for preserving cognitive health within this population and mitigating the risk of adverse cognitive outcomes. There are also some limitations to this study. First, data for this study were collected primarily in the form of self-report questionnaires, and thus some recall bias may be present in the study. Second, the results of this study are based on the analysis of data from a cross-sectional survey. Therefore, further research is needed to investigate the long-term effects of postoperative social engagement on cognitive functioning in glioma patients. Left and right hand dominance was also not part of the data collection in the design phase of our study, we intend to address it in future research endeavors.

## 6. Conclusion

In summary, the cognitive function of glioma patients early after surgery is related to social participation. The degree of social participation of patients can predict the cognitive function status in the early postoperative period to a certain extent. Actively constructing a social rehabilitation program for glioma patients and improving the level of social participation early after surgery are conducive to the protection of cognitive function of patients.

## Supporting information

S1 Dataset Supplementary Data. (SAV)
